# Impact of angiotensin-converting enzyme inhibitors versus angiotensin receptor blockers on clinical outcomes in hypertensive patients with acute myocardial infarction

**DOI:** 10.1371/journal.pone.0281460

**Published:** 2023-03-09

**Authors:** Jae-Geun Lee, Seung-Jae Joo, Song-Yi Kim, Joon-Hyouk Choi, Ki Yung Boo, Jin-Yong Hwang, Seung-Ho Hur, Myung Ho Jeong, on behalf of the KAMIR-NIH investigators

**Affiliations:** 1 Department of Internal Medicine, Jeju National University College of Medicine, Jeju, Republic of Korea; 2 Department of Internal Medicine, Jeju National University Hospital, Jeju, Republic of Korea; 3 Department of Internal Medicine, Gyeonsang National University College of Medicine, Gyeongsang National University Hospital, Jinju, Republic of Korea; 4 Keimyung University Dongsan Medical Center, Cardiovascular Medicine, Daegu, Republic of Korea; 5 Department of Internal Medicine, Chonnam National University Hospital, Gwangju, Republic of Korea; Scuola Superiore Sant’Anna, ITALY

## Abstract

There has been a concern that angiotensin receptor blockers (ARB) may increase myocardial infarction (MI) in hypertensive patients compared with other classes of anti-hypertensive drugs. Angiotensin-converting enzyme inhibitor (ACEI) is recommended as a first-line inhibitor of renin-angiotensin system (RASI) in patients with acute MI (AMI), but ARB is also frequently used to control blood pressure. This study investigated the association of ARB vs. ACEI with the long-term clinical outcomes in hypertensive patients with AMI. Among patients enrolled in the nationwide AMI database of South Korea, the KAMIR-NIH, 4,827 hypertensive patients, who survived the initial attack and were taking ARB or ACEI at discharge, were selected for this study. ARB therapy was associated with higher incidence of 2-year major adverse cardiac events, cardiac death, all-cause death, MI than ACEI therapy in entire cohort. After propensity score-matching, ARB therapy was still associated with higher incidence of 2-year cardiac death (hazard ratio [HR], 1.60; 95% confidence interval [CI], 1.20–2.14; *P* = 0.001), all-cause death (HR, 1.81; 95% CI, 1.44–2.28; *P* < 0.001), and MI (HR, 1.76; 95% CI, 1.25–2.46; *P* = 0.001) than the ACEI therapy. It was concluded that ARB therapy at discharge in hypertensive patients with AMI was inferior to ACEI therapy with regard to the incidence of CD, all-cause death, and MI at 2-year. These data suggested that ACEI be a more appropriate RASI than ARB to control BP in hypertensive patients with AMI.

## Introduction

The renin–angiotensin system (RAS) plays an important role in the development of hypertension and is also associated with the pathogenesis and progression of atherosclerosis, leading to cardiovascular (CV) disease such as myocardial infarction (MI) [1]. Angiotensin-converting enzyme inhibitors (ACEI) and angiotensin receptor blockers (ARB) are recommended as important drugs for lowering blood pressure (BP) [2]. The use of ACEI is also recommended in patients with ST-segment elevation myocardial infarction (STEMI) and non-STEMI (NSTEMI), when they have anterior infarction, heart failure (HF), left ventricular (LV) systolic dysfunction, or diabetes mellitus (DM), unless contraindicated. ARB therapy is an alternative to ACEI therapy for patients with acute MI (AMI) who are intolerant to ACEI [3, 4].

Numerous studies demonstrated the beneficial role of ACEI in patients with AMI, and ARB was non-inferior to ACEI [5–7]. Nowadays, ARB is increasingly used in patients with hypertension, HF, diabetic nephropathy, and other clinical conditions [8], and its use is not limited to subjects who complain of side-effects of ACEI such as cough or angioedema. Unfortunately, the “ARB-MI paradox” was suggested after the Valsartan Antihypertensive Long-term Use Evaluation (VALUE) trial [9], a study comparing the efficacy of valsartan with the calcium-channel blocker (CCB), amlodipine, in patients with hypertension. Despite the same degree of BP lowering, valsartan was associated with a significantly higher risk of fatal and nonfatal MI when compared with amlodipine. And given that ACEI had been shown to reduce CV events, including MI, it has been argued that ARB may increase the risk of MI.

Nevertheless, it is unclear whether ARB increases the recurrence of MI compared with ACEI in hypertensive patients after AMI. Therefore, we conducted the study to compare the clinical outcomes between ARB and ACEI treatment in hypertensive patients with AMI.

## Methods

### Study population and data collection

The study population was selected from the Korean Acute Myocardial Infarction Registry-National Institutes of Health (KAMIR-NIH) [10]. KAMIR-NIH is a nation-wide, prospective, multicenter, web-based observational cohort study aiming to develop a prognostic and surveillance index for patients with AMI. Patients who were hospitalized primarily for AMI and signed informed consents were consecutively enrolled from November 2011 to October 2015. This study was conducted according to the ethical guidelines of the Declaration of Helsinki. The study protocol was approved by the ethics committee at Chonnam National University Hospital, Republic of Korea (IRB No. CNUH-2011-172) and the institutional review boards of all participating hospitals approved the study protocol. Written informed consents were obtained from participating patients or legal representative. Data were collected by the attending physician with the assistance of a trained clinical research coordinator, via a web-based case report form in the clinical data management system of the Korea NIH. Patients, who died during index hospitalization, did not have hypertension, were prescribed neither ACEI nor ARB, or both ACEI and ARB at discharge, did not undergo echocardiographic study, and had incomplete clinical data, were excluded.

AMI was diagnosed when there was an evidence of myocardial necrosis (a rise and/or fall in cardiac biomarker, preferably cardiac troponin), and at least one of the following: (1) symptoms of ischemia, (2) new or presumed new significant ST-segment-T wave changes or a new left bundle branch block, (3) a development of pathologic Q waves in the electrocardiogram, (4) an imaging evidence of the new loss of viable myocardium or new regional wall motion abnormality, and (5) the identification of an intracoronary thrombus by angiography [11]. Hypertension was defined as values ≥140 mmHg of systolic BP (SBP) and/or ≥90 mmHg of diastolic BP (DBP) during the initial hospitalization [12, 13]. Patients with a history of hypertension or antihypertensive treatment on the interview were also considered to have hypertension. Coronary reperfusion included reperfusion by percutaneous coronary intervention (PCI), thrombolysis, or coronary artery bypass graft (CABG), MI with non-obstructed coronary arteries (MINOCA) [3], and myocardial bridge. LV systolic function was evaluated by the echocardiographic study during the initial hospitalization.

### Clinical endpoints and definition

The primary clinical endpoint was the occurrence of major adverse cardiac events (MACE), which was a composite of cardiac death (CD), MI, revascularization, and re-admission due to HF during the 2-year follow-up period. Although the recurrence of MI was the main focus, it was a secondary endpoint in this study because the primary endpoint of the KAMIR-NIH study was defined as MACE [10]. Other secondary endpoints were CD, revascularization, re-admission due to HF, all-cause death, stroke, stent thrombosis, 2-year major adverse cardiac and cerebrovascular events (MACCE) which was a composite of the primary endpoint and stroke, and 2-year MACE with non-cardiac death (NCD).

All deaths were considered to be associated with cardiac problems, unless a definite non-cardiac cause was established. Revascularization included repeated PCI or CABG on either target or non-target vessels. The staged PCI was excluded from revascularization.

The clinical follow-ups were routinely performed by visiting the hospital at 6-, 12-, 24-, and 36-month and whenever any clinical events occurred. If patients did not visit the hospitals, the outcome data were assessed by telephone interview. Clinical events were not centrally adjudicated. The physician identified all events and the principal investigator of each hospital confirmed them.

### Statistical analysis

For continuous variables, data were expressed as mean ± standard deviation or median (interquartile range) and differences between the two groups were evaluated using the unpaired t-test or Mann-Whitney U test. For discrete variables, differences were expressed as counts and percentages and were analyzed with the χ2 test between the two groups. To adjust for any potential confounders, propensity score-matching (PSM) analysis was performed using the logistic regression model with all available variables that could be of potential relevance: age, gender, body mass index (BMI), history of smoking, Killip class on admission, BP, heart rate, LV ejection fraction (LVEF), CV risk factors or co-morbidity (hypertension, diabetes mellitus, hyperlipidemia, prior HF, prior stroke, prior MI, and prior angina), initial estimated glomerular filtration rate (eGFR) by Modification of Diet in Renal Disease (MDRD) equation, co-medications (aspirin, P2Y12 inhibitors, CCB, beta-blockers and statins) at discharge and types of MI (STEMI or NSTEMI). Patients in the ARB group were 1:1 matched to those in the ACEI group according to propensity score with nearest neighbor matching algorithm. Subjects were matched with a caliper width equal to 0.1 of the standard deviation of the propensity score. The efficacy of the propensity score model was assessed by estimating standardized differences for each covariate between groups. Survival curves for clinical endpoints and cumulative event rates with incidence rates per 100 patient-years up to 2-year were generated using Kaplan–Meier estimates. Cox-proportional hazard models were used to assess the adjusted hazard ratio (HR) comparing the two groups and their 95% confidence interval (CI) for each clinical endpoint. Subgroups that were defined post-hoc according to demographic and clinical characteristics included age (<75 & ≥75 years), gender, diabetes mellitus, Killip class, LVEF (<50% & ≥50%), beta-blockers at discharge, type of MI, multi-vessel disease and infarct-related artery.

All data were processed with SPSS version 23 (IBM Co, Armonk, NY, US) and R version 3.1.3 (R Foundation for Statistical Computing, Vienna, Austria). For all analyses, a two-sided *p* < 0.05 was considered to be statistically significant.

## Results

**Fig 1:**
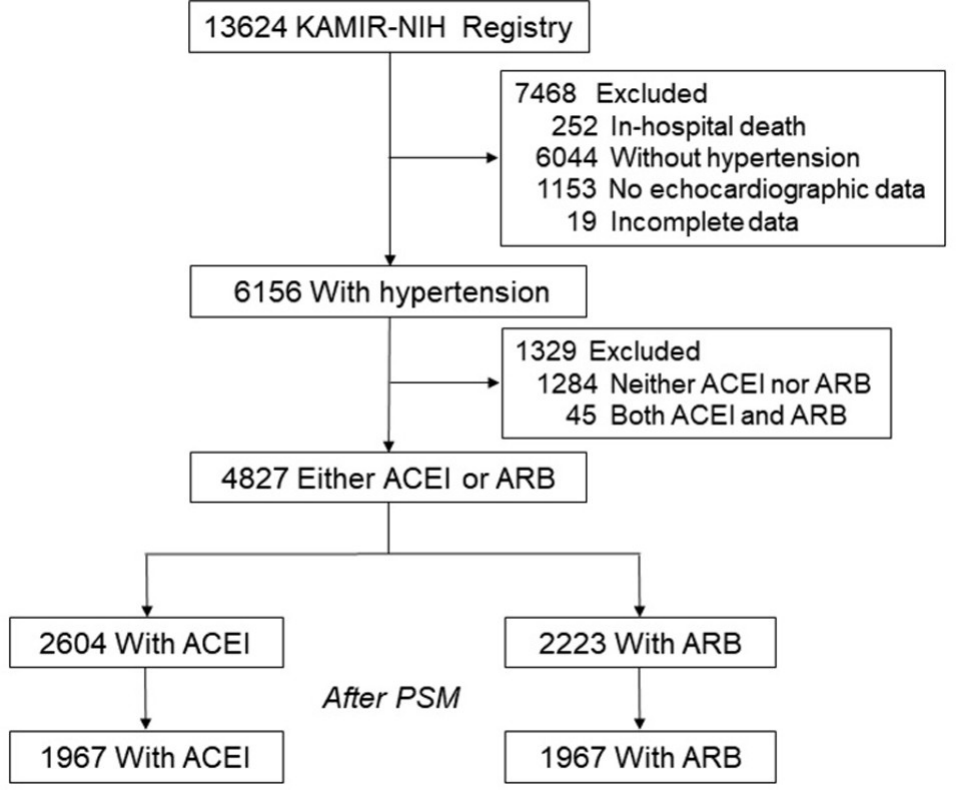
Selection of patients for analysis. ACEI, angiotensin-converting enzyme inhibitor; ARB, angiotensin receptor blocker; KAMIR-NIH, Korean Acute Myocardial Infarction Registry-National Institutes of Health; PSM, propensity score-matching.

Total 13,624 consecutive patients were enrolled in the KAMIR-NIH. After excluding 8,797 patients (252 patients who died during index hospitalization, 6,044 patients without hypertension, 1,284 patients with neither ACEI nor ARB at discharge, 45 patients with both ACEI and ARB at discharge, 1,153 patients without echocardiographic data, and 19 patients with incomplete data), 4,827 hypertensive patients with either ACEI or ARB at discharge were analyzed in this study (Fig 1). ACEI or ARB was prescribed at the discretion of attending physicians. More ACEI were used at discharge. After PSM, 1,967 patients in each group were selected.

### Baseline clinical characteristics

In the entire cohort, patients with ARB at discharge were older, and had more diabetes mellitus, prior MI, prior angina, prior HF and eGFR <60 mL/min/1.73m^2^ compared to those with ACEI (Table 1). On the other hand, patients with ACEI at discharge were more male, more current smoker, and had more STEMI, more treated with P2Y12 inhibitors or beta-blockers at discharge compared to those with ARB. The baseline LVEF of ARB group was higher than that of ACEI group. After PSM, these baseline differences between two groups were well balanced (Table 1). Overall reperfusion rate was 95%, and PCI with drug-eluting stents was the main method of coronary reperfusion in the entire and PSM cohorts.

**Table 1:** Baseline characteristics and medications at discharge.

	Entire cohort				Propensity score-matched patients			
Variables	ACEI	ARB	P value	SD	ACEI	ARB	P value	SD
	(n = 2604)	(n = 2223)			(n = 1967)	(n = 1967)		
Age, years	65.7 ± 12.0	67.5 ± 11.4	<0.001	0.16	66.9 ± 11.5	67.1 ± 11.5	0.650	0.02
Male	1817 (69.8)	1406 (63.2)	<0.001	-0.14	1305 (66.3)	1290 (65.6)	0.638	-0.02
SBP at admission	135.2 ± 28.2	133.4 ± 27.5	0.028	-0.14	132.7 ± 29.4	132.4 ± 28.5	0.546	-0.02
DBP at admission	80.3 ± 16.6	80.5 ± 16.3	0.668	0.05	79.4 ± 17.1	80.1 ± 17.3	0.296	0.03
Killip class ≥ II	532 (20.4)	493 (22.2)	0.148	0.04	414 (21.0)	429 (21.8)	0.586	0.02
Body mass index, kg/m^2^	24.3 ± 3.3	24.3 ± 3.6	0.580	0.02	24.3 ± 3.3	24.3 ± 3.6	0.798	0.01
Current smoker	879 (33.8)	594 (26.7)	<0.001	-0.16	559 (28.4)	553 (28.1)	0.859	-0.03
Diabetes mellitus	847 (32.5)	932 (41.9)	<0.001	0.19	747 (38.0)	768 (39.0)	0.512	0.01
Dyslipidemia	368 (14.1)	305 (13.7)	0.708	-0.01	276 (14.0)	275 (14.0)	>0.999	-0.01
Prior MI	191 (7.3)	238 (10.7)	<0.001	0.11	173 (8.8)	181 (9.2)	0.697	0.01
Prior angina pectoris	249 (9.6)	327 (14.7)	<0.001	0.15	231 (11.7)	256 (13.0)	0.245	0.03
Prior heart failure	40 (1.5)	53 (2.4)	0.036	0.06	38 (1.9)	43 (2.2)	0.654	0.02
Prior stroke	248 (9.5)	209 (9.4)	0.921	0.00	191 (9.7)	189 (9.6)	0.957	0.02
eGFR<60 mL/min/1.73m^2^	563 (21.6)	611 (27.5)	<0.001	0.13	481 (24.5)	501 (25.5)	0.484	0.02
LVEF, %	51.2 ± 10.9	53.8 ± 11.3	<0.001	0.22	52.8 ± 10.6	53.1 ± 11.2	0.504	0.02
STEMI	1316 (50.5)	810 (36.4)	<0.001	-0.29	814 (41.4)	793 (40.3)	0.517	-0.02
Coronary reperfusion^a^	2479 (95.2)	2115 (95.1)	0.946	0.00	1876 (95.4)	1873 (95.2)	0.880	-0.03
SBP at discharge	116.9 ± 15.1	115.9 ± 16.0	0.036		117.3 ± 17.1	115.5 ± 15.7	0.001	
DBP at discharge	69.9 ± 10.2	69.0 ± 10.1	0.002		70.0 ± 10.3	68.9 ± 10.1	0.002	
Medications at discharge								
Aspirin	2601 (99.9)	2219 (99.8)	0.710	-0.02	1964 (99.8)	1963 (99.8)	>0.999	-0.01
P2Y12 inhibitors	2544 (97.7)	2124 (95.5)	<0.001	-0.10	1911 (97.2)	1906 (96.9)	0.708	-0.01
Beta-blockers	2390 (91.8)	1914 (86.1)	<0.001	-0.16	1762 (89.6)	1737 (88.3)	0.222	-0.04
Statins	2476 (95.1)	2093 (94.2)	0.158	-0.04	1967 (100.0)	1967 (100.0)	>0.999	-0.01

Values are mean ± standard deviation or number (%).

ACEI, angiotensin-converting enzyme inhibitor; ARB, angiotensin receptor blocker; DBP, diastolic blood pressure; eGFR, estimated glomerular filtration rate; LVEF, left ventricular ejection fraction; MI, myocardial infarction; SBP, systolic blood pressure; SD, standardized difference; STEMI, ST-elevation myocardial infarction.

^a^Included reperfusion by percutaneous coronary intervention, thrombolysis, or coronary artery bypass graft, myocardial infarction with non-obstructed coronary arteries, and myocardial bridge.

### Clinical outcomes

Two-year follow-up rate was 94% and 97% in the entire and PSM cohorts, respectively. In the entire cohort, 43% of patients with ACEI at discharge continued to take ACEI at 1-year, but 36% had cross-over to ARB. On the other hand, 82% of patients with ARB at discharge continued to take ARB, and only 1.4% had cross-over to ACEI. Also, at 2-year, 34% of patients with ACEI at discharge continued to take ACEI, and 38% had cross-over to ARB. Among patients with ARB at discharge, 70% of patients continued to take ARB, and only 1.3% had cross-over to ACEI. Cross-over rates in PSM cohort showed a similar pattern.

**Fig 2:**
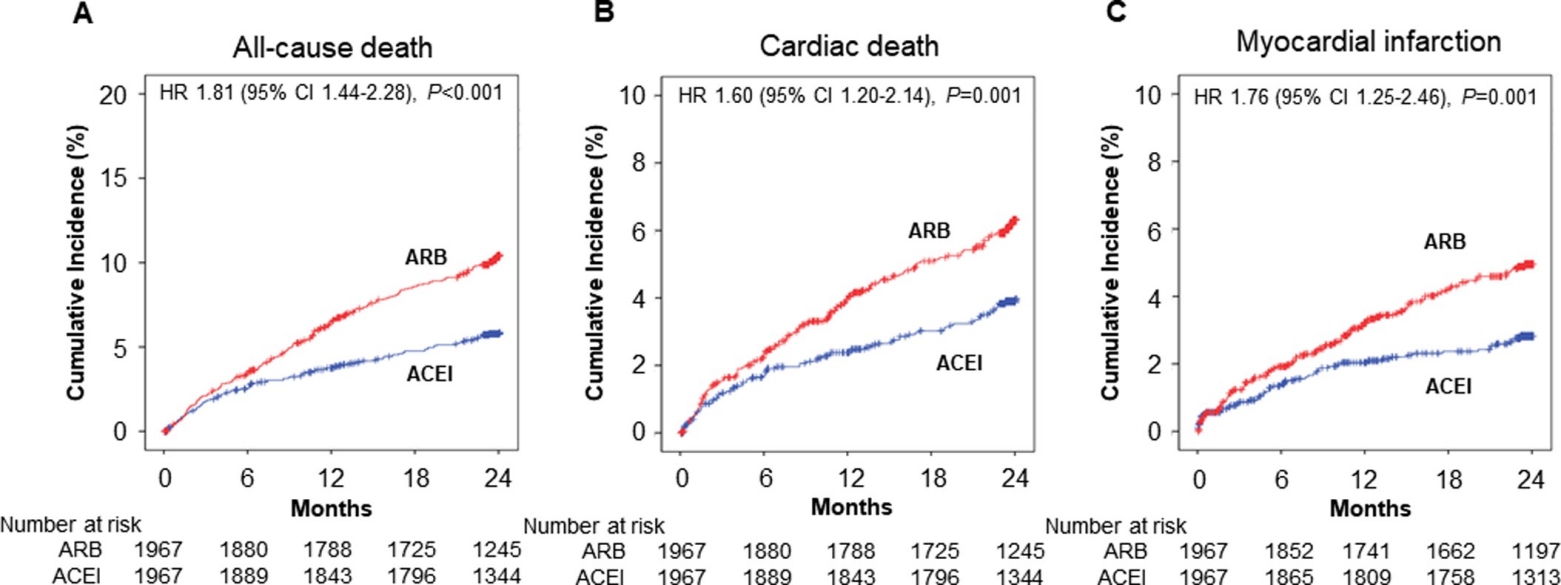
Kaplan-Meier curves and adjusted hazard ratios for 2-year clinical events in propensity score-matched patients with ARB vs. ACEI. (A) All-cause death. (B) Cardiac death. (C) Myocardial infarction. ACEI, angiotensin converting enzyme inhibitor; ARB, angiotensin receptor blocker; CI, confidence interval; HR, hazard ratio.

In entire cohort, the ARB therapy at discharge was associated with higher incidence of MACE, CD, all-cause death, MI, Stroke, MACCE and MACE with NCD at 2-year than the ACEI therapy at discharge (Table 2). However, there was no significant difference in the incidence of revascularization, re-hospitalization due to HF and stent thrombosis between two groups. After PSM, the ARB therapy at discharge was still associated with higher incidence of CD (HR, 1.60; 95% CI, 1.20–2.14; *P* = 0.001), all-cause death (HR, 1.81; 95% CI, 1.44–2.28; *P*<0.001), MI (HR, 1.76; 95% CI, 1.25–2.46; *P* = 0.001), Stroke (HR, 1.97; 95% CI, 1.26–3.09; *P* = 0.003), MACCE (HR, 1.20; 95% CI, 1.04–1.39; *P* = 0.015) and MACE with NCD (HR, 1.22; 95% CI, 1.05–1.41; *P* = 0.008) than the ACEI therapy at discharge (Table 2, Fig 2). Likewise, 1-year CD, all-cause death, MI, Stroke, MACCE and MACE with NCD were significantly higher in patients with the ARB therapy at discharge in entire and PSM cohorts (S1 Table).

**Table 2:** Multivariate Cox-proportional hazard ratio analysis of 2-year clinical events.

Outcomes	ACEI	ARB	Hazard ratio^a^ (95% CI)	*P* value
	No. of patients with events (Rate per patient-years)			
**Entire cohort**	(n = 2604)	(n = 2223)		
MACE	383 (8.5)	401 (10.7)	1.25 (1.09–1.44)	0.002
Cardiac death	93 (1.9)	143 (3.5)	1.84 (1.41–2.38)	<0.001
All-cause death	137 (2.8)	232 (5.7)	2.02 (1.64–2.50)	<0.001
Myocardial infarction	66 (1.4)	108 (2.7)	1.96 (1.44–2.67)	<0.001
Revascularization	206 (4.5)	175 (4.5)	1.01 (0.83–1.24)	0.898
Heart failure^b^	107 (2.3)	105 (2.7)	1.16 (0.89–1.52)	0.277
Stroke	36 (0.8)	61 (1.5)	2.03 (1.35–3.07)	0.001
Stent thrombosis	12 (0.3)	15 (0.4)	1.49 (0.70–3.18)	0.306
MACCE	414 (9.3)	444 (12.0)	1.29 (1.13–1.47)	<0.001
MACE with non-cardiac death	421 (9.4)	477 (12.8)	1.26 (1.10–1.44)	0.001
**Propensity score-matched cohort**	(n = 1967)	(n = 1967)		
MACE	304 (9.0)	346 (10.5)	1.16 (0.99–1.35)	0.063
Cardiac death	75 (2.0)	118 (3.3)	1.60 (1.20–2.14)	0.001
All-cause death	112 (3.1)	199 (5.6)	1.81 (1.44–2.28)	<0.001
Myocardial infarction	53 (1.5)	91 (2.6)	1.76 (1.25–2.46)	0.001
Revascularization	162 (4.6)	158 (4.6)	0.99 (0.80–1.24)	0.948
Heart failure^b^	87 (2.4)	89 (2.5)	1.03 (0.77–1.39)	0.830
Stroke	29 (0.8)	56 (1.6)	1.97 (1.26–3.09)	0.003
Stent thrombosis	10 (0.3)	14 (0.4)	1.42 (0.63–3.20)	0.395
MACCE	329 (9.8)	387 (11.8)	1.20 (1.04–1.39)	0.015
MACE with non-cardiac death	337 (9.9)	414 (12.5)	1.22 (1.05–1.41)	0.008

ACEI, angiotensin-converting enzyme inhibitor; ARB, angiotensin receptor blocker; CI, confidence interval; MACCE, major adverse cardiocerebral event; MACE, major adverse cardiac event.

^a^Adjusted for age, sex, body mass index, diabetes mellitus, dyslipidemia, prior angina, prior myocardial infarction, prior heart failure, current smoker, Killip class, estimated glomerular filtration rate, left ventricular ejection fraction, type of myocardial infarction, coronary reperfusion, and medications (aspirin, P2Y12 inhibitors, BB, and statins) at discharge.

^b^Re-hospitalization due to heart failure.

**Fig 3:**
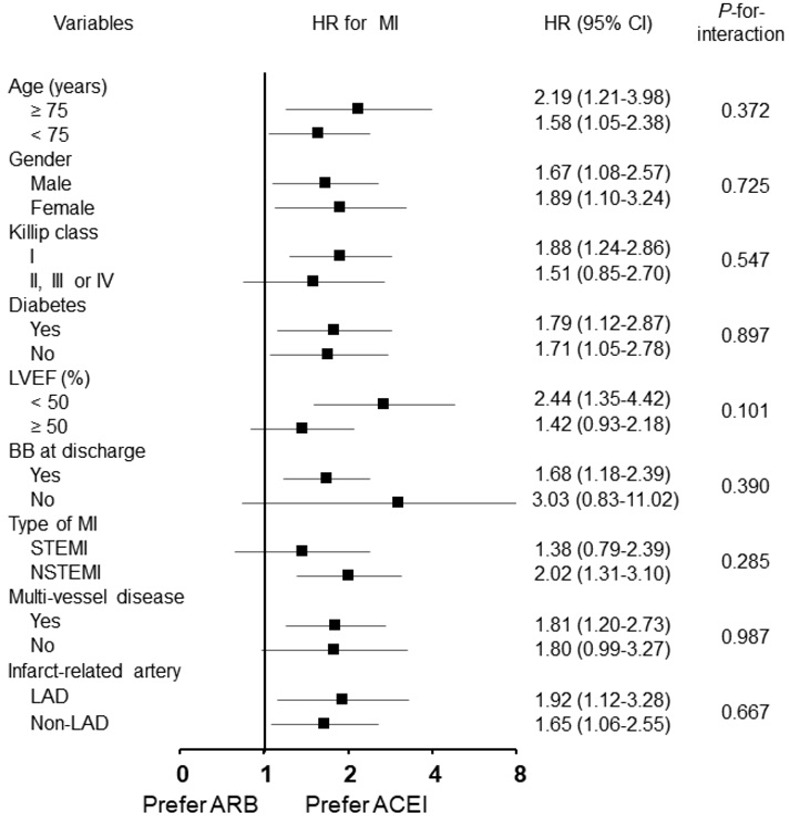
Subgroup analysis for myocardial infarction in propensity score-matched patients with ARB vs. ACEI. ACEI, angiotensin converting enzyme inhibitor; ARB, angiotensin receptor blocker; CI, confidence interval; HR, hazard ratio; MI; myocardial infarction; NSTEMI, non-ST elevation myocardial infarction; LAD, left anterior descending artery; LVEF, left ventricular ejection fraction; STEMI, ST-elevation myocardial infarction.

Compared with ACEI therapy, the inferior association between the ARB therapy at discharge and 2-year MI appeared to be consistent across a series of subgroups, including age, gender, diabetes mellitus, Killip class, LVEF, beta-blockers at discharge, and type of MI (Fig 3). In PSM cohort with reduced LVEF (<50%), ARB therapy at discharge was associated with a significantly higher incidence of CD, all-cause death, MI, and MACE with NCD at 2-year than the ACEI therapy at discharge. On the other hand, in PSM cohort with preserved LVEF (≥50%), the incidence of MI was not different between the ACEI and ARB therapy, but the incidence of all-cause death, CD, stroke and MACCE was higher in ARB group (S2 Table and S1 Fig).

In propensity score-matched cohort, BP at discharge in ARB group were lower than that in ACEI group (SBP; 115.5 ± 15.7mmHg, vs. 117.3±15.5mmHg; *P* = 0.001, DBP; 68.9±10.1mmHg vs. 70.0±10.3mmHg; *P* = 0.002), however, BP at the admission, 1- and 2-year were not different (S2 Fig).

**Fig 4:**
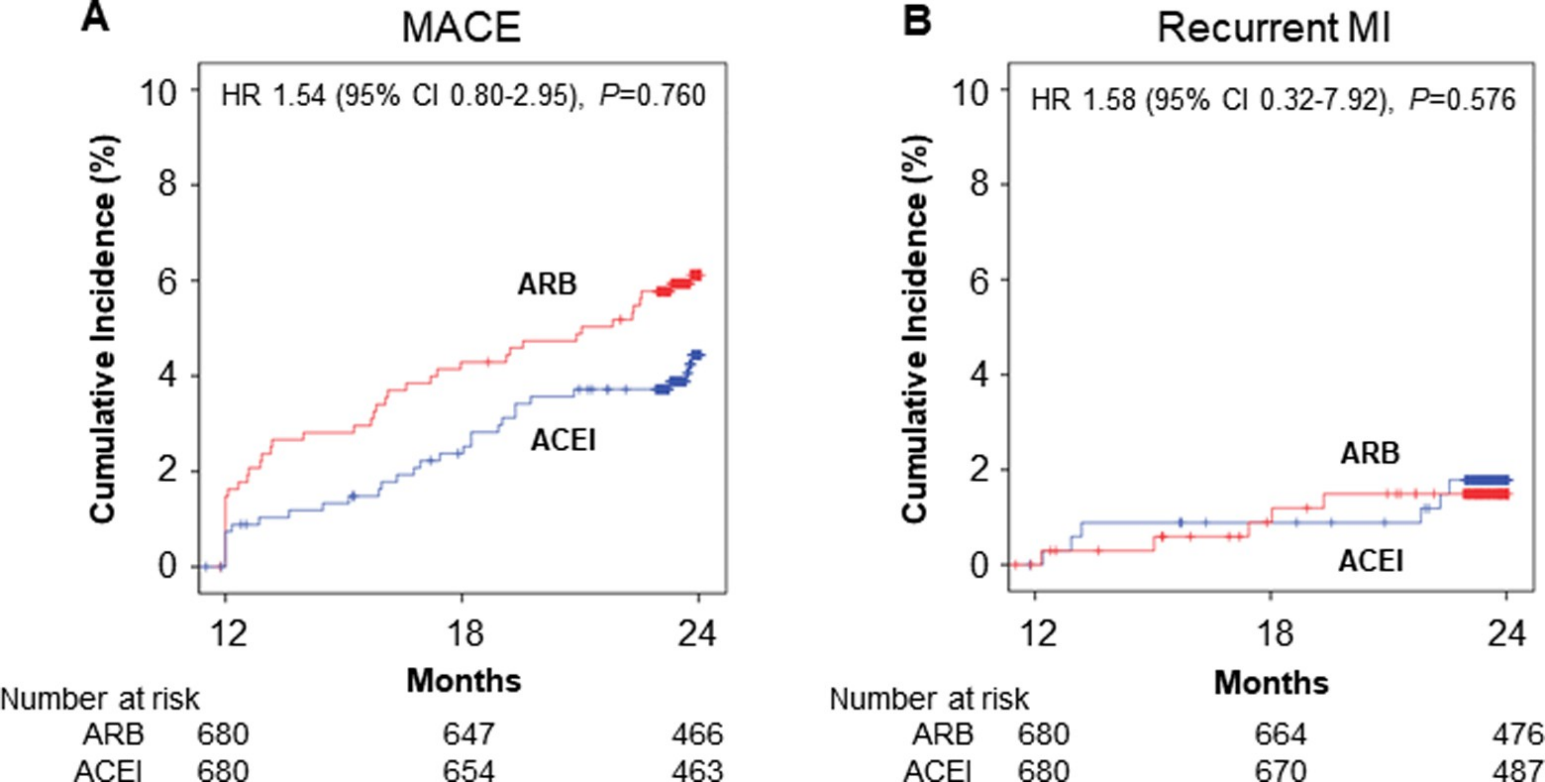
Landmark analysis for MACE and recurrent MI among patients who were event-free at 1-year after propensity score matching. ACEI, angiotensin converting enzyme inhibitor; ARB, angiotensin receptor blocker; CI, confidence interval; HR, hazard ratio, MACE, major adverse cardiac events; MI, myocardial infarction.

We performed 1-year landmark analysis for the incidence of clinical events from 1 to 2-year among patients who were event-free at 1-year. The number of stable patients who were event-free at 1-year was 4,174 out of total 4,827 patients. At 1-year follow-up, 3,547 patients were taking RAS inhibitors (RASI). After excluding 41 patients taking both ACEI and ARB, 2,444 patients were taking ARB and 1,062 patients were taking ACEI. After PSM, 680 patients in each group were selected. The incidence of MACE and recurrent MI at 2-year was not statistically different, indicating that there was no difference between ARB and ACEI in clinical events in patients who were relatively stable after AMI (Fig 4).

**Fig 5:**
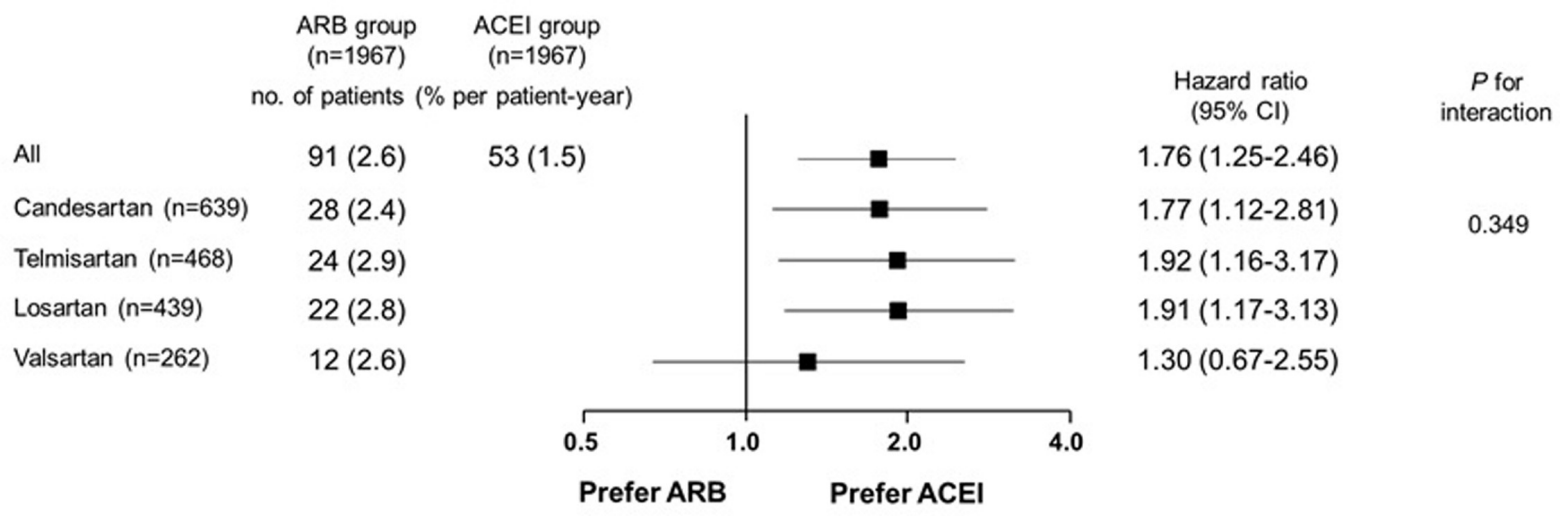
Adjusted hazard ratios of 2-year recurrent myocardial infarction in propensity score-matched cohort with ARB vs. ACEI according to generic names of ARB. ACEI, angiotensin converting enzyme inhibitor; ARB, angiotensin receptor blocker; CI, confidence interval.

Perindopril (50%) and ramipril (40%) were the major ACEI’s, and candesartan (35%), losartan (24%), telmisartan (20%) and valsartan (14%) were the major ARB’s that prescribed at discharge (Table 3). All RASI were used in lower doses than those recommended in the guidelines. ARB’s association with higher incidence of 2-year MI than ACEI was consistent across the generic names of ARB’s without a significant interaction (Fig 5).

**Table 3:** Generic names and doses of renin-angiotensin system inhibitors that prescribed at discharge in propensity-score matched cohort.

Generic name	No. of patients	Mean dose (mg)	Median dose (mg)
Angiotensin-converting enzyme inhibitors (n = 1967)			
Perindopril	998 (50.7)	3.1±1.5	2.0 (2.0–4.0)
Ramipril	832 (42.3)	2.9±3.0	2.5 (1.3–2.5)
Captopril	84 (4.3)	26.0±29.6	18.8 (9.4–37.5)
Others	53 (2.7)		
Angiotensin receptor blockers (n = 1967)			
Candesartan	639 (32.5)	8.0±4.8	4 (4–8)
Telmisartan	468 (23.8)	46.0±19.8	40 (40–40)
Losartan	439 (22.3)	45.7±21.0	50 (25–50)
Valsartan	262 (13.3)	96.2±67.4	80 (40–160)
Others	159 (8.1)		

Values are number (%), mean±standard deviation, or median (interquartile range).

## Discussion

The main findings of this study are that ARB therapy at discharge was inferior to ACEI therapy with regard to the incidence of CD, and all-cause death, and MI in patients with hypertension and AMI, up to 2-year of follow-up. In patients who had no clinical events until 1-year, there was no difference in the incidence of clinical events between ACEI and ARB therapy at 2-year follow-up.

In other cohort studies [14, 15], approximately 46~67% of patients with AMI received RASI. In this study, 78% of hypertensive subjects with AMI received either ACEI or ARB at discharge, and ARB was prescribed in 46% of those patients; this result reflects the “real world” practice in hypertensive patients with AMI. In patients with AMI, optimal medical therapy plays an important role for secondary prevention, and RASI is one of the important drugs when they have hypertension. ACEI is recommend as a first-line RASI for hypertensive patients with AMI [16], and when they are intolerant to ACEI, ARB is an alternative RASI to be prescribed at discharge. However, despite this recommendation, ARB is occasionally used as the first line RASI because ARB has an advantage of better tolerability than ACEI.

There has been a long-standing debate that ARB has less preventive effects on all-causes death, CV death, and CV events than ACEI [17, 18]. There were meta-analyses that focused on this issue [19, 20]. In patients with diabetes mellitus or without HF, ACEI reduced all-cause death, CV death, and MI when compared with either active drugs or placebo, but ARB showed no benefits for these outcomes. However, the control event rate, which affected the efficacy of RASI therapy, has been lowered since 2000 because of more wide use of statin therapy and strict BP control in hypertensive patients. The relatively lower control event rate of the major ARB trials which were performed after 2000 may explain the lack of clinical benefits of ARB. Indeed, the meta-analysis of head-to-head comparison trials showed similar clinical outcomes between ARB and ACEI [19].

In patients with AMI, the clinical trial and observational studies comparing relative efficacy of ACEIs and ARBs on long-term clinical outcomes showed inconsistent results. In the Valsartan in Acute Myocardial Infarction trial which compared valsartan and captopril in MI patients with HF or LV systolic dysfunction, valsartan was not inferior to captopril in reducing the incidence of all-cause death, cardiac death, and MI [6, 21] and one registry data of patients with AMI showed that ACEI and ARB had similar risks of cardiac death or MI up to 1-year follow-up [22]. However, other observational studies of patients with AMI showed that ARB was inferior to ACEI in reducing all-cause death, MACE or any repeat revascularization [23–25]. In a recent registry study of patients with AMI without a history of hypertension [26], ACEI therapy was associated with reduced incidences of MACE, any repeat revascularization, stroke, and re-hospitalization due to HF than ARB therapy, but MI was not significantly different between ACEI and ARB therapy. In our study of hypertensive patients with AMI, the cumulative incidences of cardiac death, all-cause death, and MI at 2-year was higher in ARB group than in the ACEI group, but in patients without events until 1-year, clinical outcomes were not different between the two groups. These findings suggest that ACEI in hypertensive patients with AMI is associated with better clinical outcomes in the initial period compared with ARB, but after stabilization from the acute attack at 1-year, clinical outcomes are similar regardless of which RASI is administered.

There are plausible mechanisms about “ARB-MI paradox”. ACEI inhibits the formation angiotensin II (Ang II) to prevent its pathological effects on endothelial function, CV remodeling, and the progression of atherosclerosis. ACEI also prevents the breakdown of bradykinin, resulting in additional cardioprotective effects. However, ARB selectively blocks Ang II type 1 receptors, which leads to a marked counter up-regulation of Ang II. The augmented stimulation of Ang II type 2 receptor was shown to promote the release of leukocyte-dependent matrix metalloproteinase-1 and resultant atherosclerotic plaque rupture. It may also lead to apoptosis and inhibition of angiogenesis which have a potential to decrease collateral vessel growth even in ischemic conditions [27]. These mechanisms may explain the superiority of ACEI over ARB in reducing MACE, CV death, MI, revascularization, and re-hospitalization due to HF in patients with AMI.

## Limitations

This study has several limitations. First, this study analyzed a non-randomized, observational registry data. The prescription and selection of RASI was at the discretion of an attending physician. The information why physicians prescribed ACEI or ARB at discharge was not available. Although we performed a PSM analysis to account for the potential confounding factors, other unmeasured, residual variables as well as selection bias could not be completely controlled. However, a randomized clinical trial of head-to-head comparison between ACEI and ARB in hypertensive patients with AMI is very difficult to be performed. In this respect, observational registry data may answer which RASI has better clinical outcomes despite the inherent limitations. Second, because patients’ medications were recorded only at discharge, 1-year and 2-year, we could not ascertain whether patients actually obtained them, took them as prescribed, and adhered for two years. In addition, a large cross-over was observed in patients with ACEI or ARB during 2 years. However, taking ACEI from the hospital discharge was associated with better clinical outcomes than ARB. Third, the clinical events were not centrally adjudicated, but instead, identified by an attending physician and confirmed by the principal investigator of each hospital. As a result, some clinical events may not have been captured in the database. Fourth, 2-year follow-up may not be long enough to evaluate clinical association of ARB with MI.

## Conclusions

ARB therapy at discharge in hypertensive patients with AMI who survived the initial attack was inferior to ACEI therapy with regard to the incidence of CD, all-cause death, and MI at 2-year. These data suggested that ACEI be a more appropriate RASI to control BP in hypertensive patients with AMI.

## Supporting information

S1 Fig Kaplan-Meier curves and adjusted hazard ratios for 2-year clinical events in the propensity score-matched cohort with ARB vs. ACEI according to left ventricular ejection fraction. (A) MACE in patients with LVEF <50%. (B) MACE in patients with LVEF ≥50%. (C) All-cause death in patients with LVEF <50%. (D) All-cause death in patients with LVEF ≥50%. (E) Cardiac death in patients with LVEF <50%. (F) Cardiac death in patients with LVEF ≥50%. (G) Myocardial infarction in patients with LVEF <50%. (H) Myocardial infarction in patients with LVEF ≥50%. ACEI, angiotensin converting enzyme inhibitor; ARB, angiotensin receptor blocker; CI, confidence interval; HF, heart failure; HR, hazard ratio; MACE, major adverse cardiac events; MI, myocardial infarction.
(TIF)

S2 Fig Blood pressure at admission, discharge, 1-year, and 2-year in propensity score-matched cohort with ARB vs. ACEI. (A) Systolic blood pressure. (B) Diastolic blood pressure. ACEI, angiotensin converting enzyme inhibitor; ARB, angiotensin receptor blocker.
(TIF)

S1 Table Multivariate Cox-proportional hazard analysis of 1-year clinical events. (DOCX)

S2 Table Multivariate Cox-proportional hazard analysis of 2-year clinical events according to left ventricular ejection fraction in propensity-score matched cohort. (DOCX)

S1 Dataset Minimal raw clinical data with anonymization. (XLSX)
