# Supplementary figures and images for: Impact of angiotensin-converting enzyme inhibitors versus angiotensin receptor blockers on clinical outcomes in hypertensive patients with acute myocardial infarction
Source: PLoS One. 2023 Mar 9;18(3):e0281460. doi: 10.1371/journal.pone.0281460 (PMC9997890; doi:10.1371/journal.pone.0281460)

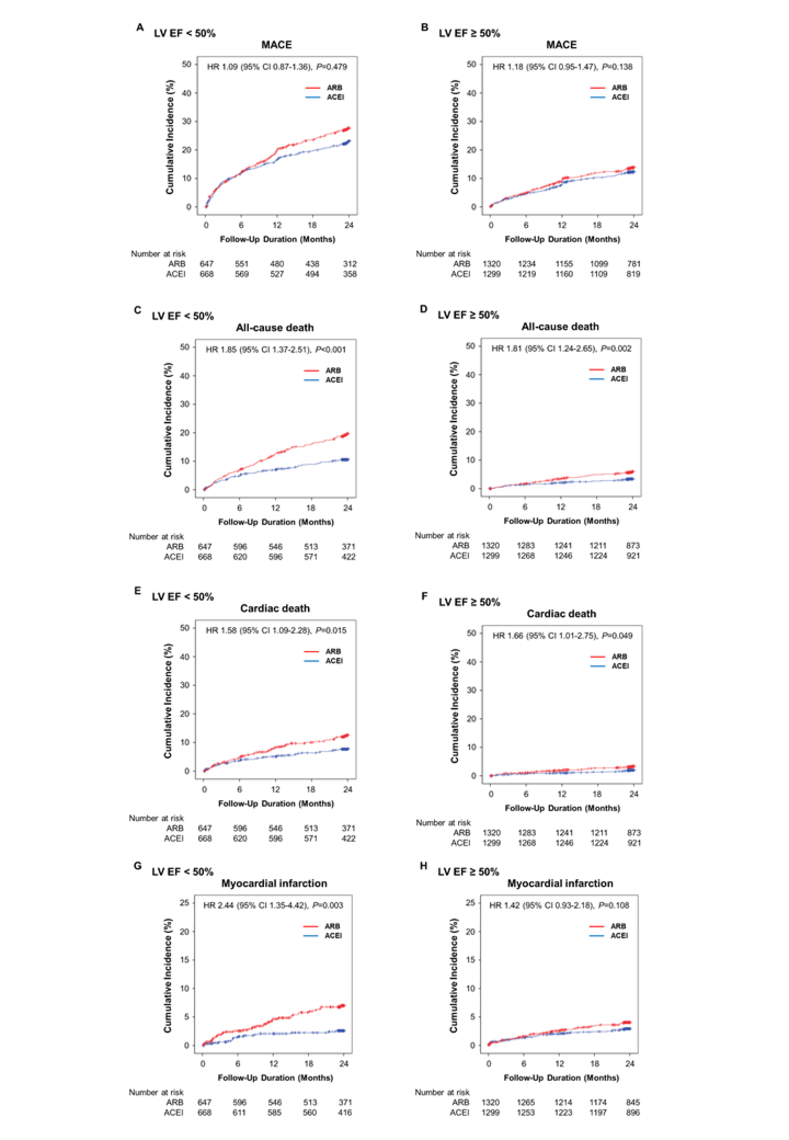

Supplement: S1 Fig — (A) MACE in patients with LVEF <50%. (B) MACE in patients with LVEF ≥50%. (C) All-cause death in patients with LVEF <50%. (D) All-cause death in patients with LVEF ≥50%. (E) Cardiac death in patients with LVEF <50%. (F) Cardiac death in patients with LVEF ≥50%. (G) Myocardial infarction in patients with LVEF <50%. (H) Myocardial infarction in patients with LVEF ≥50%. ACEI, angiotensin converting enzyme inhibitor; ARB, angiotensin receptor blocker; CI, confidence interval; HF, heart failure; HR, hazard ratio; MACE, major adverse cardiac events; MI, myocardial infarction. (TIF) [file pone.0281460.s001.tif]

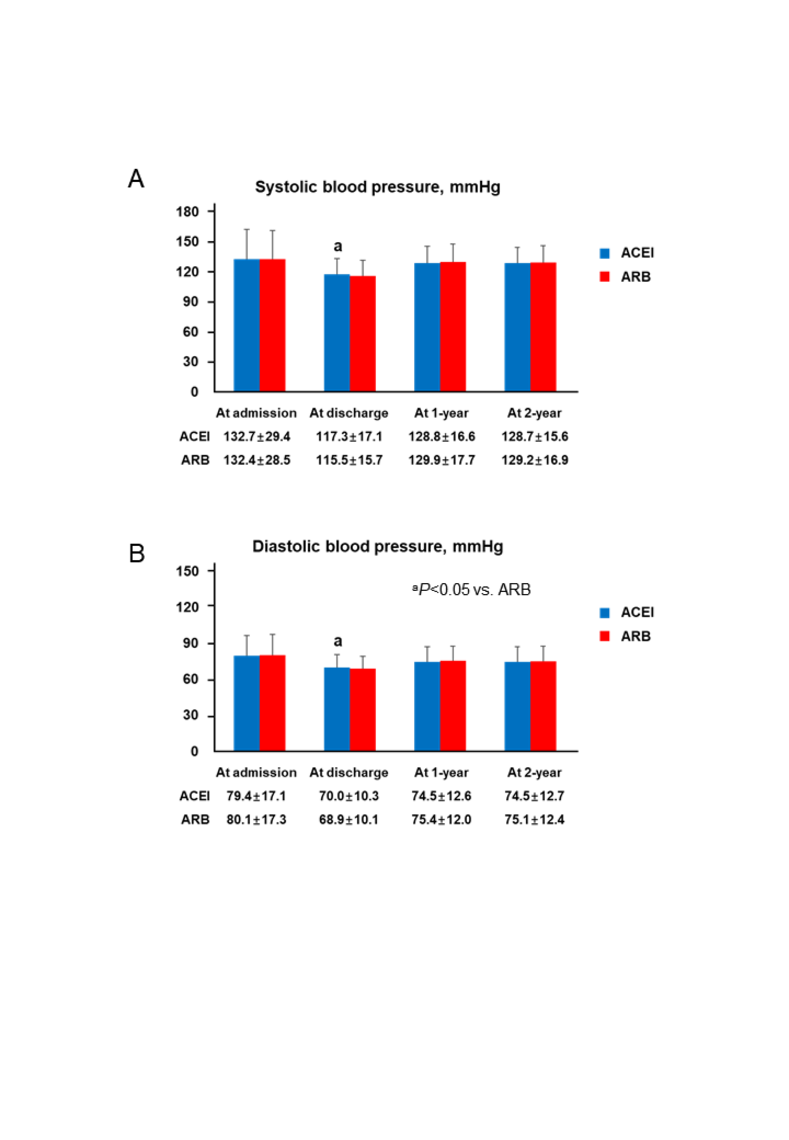

Supplement: S2 Fig — (A) Systolic blood pressure. (B) Diastolic blood pressure. ACEI, angiotensin converting enzyme inhibitor; ARB, angiotensin receptor blocker. (TIF) [file pone.0281460.s002.tif]
