# Supplementary material for: Impact of angiotensin-converting enzyme inhibitors versus angiotensin receptor blockers on clinical outcomes in hypertensive patients with acute myocardial infarction
Source: PLoS One. 2023 Mar 9;18(3):e0281460. doi: 10.1371/journal.pone.0281460 (PMC9997890; doi:10.1371/journal.pone.0281460)
Supplement: S1 Table — (DOCX) [file pone.0281460.s003.docx]

**Table S1. Multivariate Cox-proportional hazard analysis of 1-year clinical events**

| Outcomes | ACEI | ARB | Hazard ratio^a^  (95% CI) | *P* value |
| --- | --- | --- | --- | --- |
|  | No. of patients with events  (Rate per 100 patient-years) | |  |  |
| **Entire cohort** | (n=2604) | (n=2223) |  |  |
| MACE | 259 (10.9) | 274 (13.6) | 1.16 (0.98-1.39) | 0.093 |
| Cardiac death | 58 (2.3) | 89 (4.2) | 1.65 (1.17-2.33) | 0.005 |
| All-cause death | 91 (3.6) | 138 (6.5) | 1.67 (1.27-2.19) | <0.001 |
| Myocardial infarction | 48 (1.9) | 72 (3.4) | 1.57 (1.08-2.29) | 0.019 |
| Revascularization | 130 (5.3) | 107 (5.2) | 0.97 (0.74-1.26) | 0.800 |
| Heart failure^b^ | 84 (3.4) | 83 (4.0) | 1.05 (0.77-1.44) | 0.770 |
| Stroke | 25 (1.0) | 43 (2.0) | 1.86 (1.12-3.09) | 0.017 |
| Stent thrombosis | 9 (0.4) | 8 (0.4) | 1.07 (0.40-2.83) | 0.897 |
| MACCE | 280 (11.8) | 308 (15.4) | 1.21 (1.02-1.43) | 0.026 |
| MACE with non-cardiac death | 288 (12.1) | 316 (15.6) | 1.21 (1.03-1.43) | 0.025 |
| **Propensity score-matched cohort** | (n=1967) | (n=1967) |  |  |
| MACE | 206 (11.5) | 241 (13.5) | 1.14 (0.95-1.38) | 0.165 |
| Cardiac death | 46 (2.4) | 77 (4.1) | 1.65 (1.14-2.40) | 0.009 |
| All-cause death | 73 (3.9) | 124 (6.6) | 1.71 (1.28-2.30) | <0.001 |
| Myocardial infarction | 39 (2.1) | 60 (3.2) | 1.58 (1.05-2.36) | 0.028 |
| Revascularization | 103 (5.6) | 100 (5.5) | 0.99 (0.75-1.30) | 0.930 |
| Heart failure^c^ | 67 (3.6) | 71 (3.9) | 1.00 (0.71-1.41) | 0.989 |
| Stroke | 21 (1.1) | 40 (2.1) | 1.95 (1.15-3.32) | 0.014 |
| Stent thrombosis | 8 (0.4) | 8 (0.4) | 1.06 (0.40-2.85) | 0.903 |
| MACCE | 224 (12.5) | 273 (15.4) | 1.20 (1.01-1.43) | 0.047 |
| MACE with non-cardiac death | 230 (12.8) | 281 (15.7) | 1.20 (1.01-1.44) | 0.046 |
| ACEI, angiotensin-converting enzyme inhibitor; ARB, angiotensin receptor blocker; CI, confidence interval; MACCE, major adverse cardiocerebral event; MACE, major adverse cardiac event.  ^a^Adjusted for age, sex, body mass index, diabetes mellitus, dyslipidemia, prior angina, prior myocardial infarction, prior heart failure, current smoker, Killip class, estimated glomerular filtration rate, left ventricular ejection fraction, type of myocardial infarction, coronary reperfusion, and medications (aspirin, P2Y12 inhibitors, calcium-channel blockers, beta-blockers, and statins) at discharge.  ^b^Re-hospitalization due to heart failure. | | | | |
